# Migratory Experience as a Factor of Vulnerability: Navigating Loss, Gratitude, and Meaning

**DOI:** 10.3390/healthcare13172109

**Published:** 2025-08-25

**Authors:** María José Cáceres-Titos, E. Begoña García-Navarro, Mayckel da Silva Barreto

**Affiliations:** 1 Department of Nursing, University of Huelva, Campus El Carmen s/n, 21007 Huelva, Spain; 2 Research Centre COIDESO, University of Huelva, 21007 Huelva, Spain; 3 Research Group ESEIS, University of Huelva, 21007 Huelva, Spain; 4 Department of Nursing, State University of Maringá, Maringá 87020-900, Brazil; msbarreto@uem.br

**Keywords:** migration, refugees, mental health, coping, culture, health, qualitative research

## Abstract

**Background/Objectives:** Involuntary migration exposes individuals to multiple losses and ruptures that profoundly affect their physical, emotional, and social well-being. This study aimed to explore the vital losses experienced by Latin American women seeking international protection, identifying key dimensions of these losses and the coping strategies they employ to support their health and well-being. **Methods:** The study employed a qualitative phenomenological approach, with 17 international migrant women comprising the study subjects. Data were analysed using an inductive approach and interpretative phenomenological analysis, facilitated by Atlas.ti 23.0 software. The COREQ criteria were followed. **Results:** The analysis revealed two central themes: the multiplicity of losses associated with migration, including loss of identity, emotional deterioration, disruption of family and community ties, economic instability, and loss of sense of belonging; and hidden gains, encompassing processes of gratitude, spiritual strength, and personal transformation. **Conclusions:** The findings highlight the complexity of both the losses and the hidden gains associated with the migration experience, underscoring the need for compassionate and culturally competent healthcare. This study provides relevant evidence to improve professional support strategies for refugee women from a comprehensive and humanised perspective.

## 1. Introduction

The migratory phenomenon, which has intensified in recent decades, is not limited to a mere geographical displacement. It involves a profound life transformation, entailing multiple significant losses that affect the emotional, physical, and symbolic well-being of migrants. This process of personal reorganisation following the loss of meaningful elements is known as “grief” [1] and is referred to as migratory grief when it relates to losses associated with the migration experience.

Migratory grief is characterised by being multiple, partial, and non-definitive [2]. This multiplicity refers to the convergence of numerous losses: family and friends (interpersonal losses) and housing and income (material losses), as well as social status and role, mother tongue, future plans, cultural identity, and sense of belonging (symbolic or ambiguous losses) [3,4,5]. The latter, being less visible and socially recognised, often remains unattended, which hinders emotional processing and may increase the risk of mental health complications if not adequately addressed [6,7].

According to the International Organization for Migration [8], a refugee is any person who, “owing to a well-founded fear of being persecuted for reasons of race, religion, nationality, membership of a particular social group or political opinion, is outside the country of their nationality and is unable, or owing to such fear, unwilling to avail themselves of the protection of that country.” Although this category entails protective rights, the migratory process itself involves a multidimensional rupture that requires thorough exploration.

Empirical evidence has shown a higher prevalence of mental disorders among forced migrants compared to those migrating voluntarily [9]. A meta-analysis by Lindert et al. [10] estimated a prevalence of 44% for depression and 40% for anxiety among refugee populations. Similarly, a systematic review by Kokou-Kpolou et al. [11] identified that 33.2% of adult refugees exhibit symptoms consistent with prolonged grief disorder. In this context, mental and emotional suffering is strongly influenced by structural risk factors such as gender, legal status, language barriers, social isolation, discrimination, and limited access to healthcare and housing [12,13,14,15,16,17].

These vulnerabilities do not arise in isolation but are rooted in systems of inequality. Nancy Krieger’s ecosocial theory [18] highlights how structural and social inequalities become biologically and emotionally embodied, creating health disparities over time. Similarly, Kimberlé Crenshaw’s *intersectionality framework* [19] provides a lens for understanding how overlapping systems of oppression shape distinct patterns of exclusion and suffering.

In Spain, migratory flows have increased significantly in recent decades, especially from Latin America, leading to a growing presence of women seeking international protection [20]. These women often arrive in contexts of high vulnerability, where institutional systems struggle to respond to their specific emotional and cultural needs [21].

From a critical perspective, understanding the losses experienced by migrant women requires moving beyond frameworks that label them solely as “vulnerable” or “excluded.” While often well-intentioned, such categories can inadvertently reinforce forms of invisibility if they are not accompanied by genuine efforts to listen to women’s voices and understand how they themselves interpret and make sense of their migratory experience. As Michel Foucault pointed out in his theory of discourse and power [22], language is not neutral but a social practice that constructs knowledge, identities, and realities. The way migrant women are named and represented shapes how they are perceived—and even how they may come to perceive themselves. For this reason, it is essential to address not only structural factors, but also the meaning-making processes through which these women articulate their own losses [23]. Listening to their narratives allows us to identify not only what they have lost, but how they live, explain, and cope with their suffering through their cultural, relational, and symbolic frameworks. Such understanding is key to avoiding the reproduction of epistemic violence and moving towards more humane, equitable, and culturally sensitive care practices.

Following this approach, various studies on psychosocial migration have shown that migrants may experience ambiguous losses [24,25], which can only be fully understood if their own narratives and interpretive frameworks are taken into account. For migrants, the physical separation from their families is compounded by the cultural dislocation they face, creating a sense of living between two worlds [24]. Several studies have documented the hope held by migrants of maintaining a psychological presence within their families, particularly in the lives of their children [26,27].

The loss of socio-relational resources is particularly significant for refugees from sociocentric and collectivist cultures, in which interpersonal relationships are fundamental to life [28]. Latin American women, in particular, tend to interpret their migration experiences through culturally specific lenses. In many of these contexts, suffering is not seen solely as an individual pathology but as a rupture of relational, moral, and spiritual ties. Loss is often expressed through relational and symbolic frameworks—such as motherhood, spirituality, or community bonds—which are rarely recognized in Western biomedical approaches [24]. The cultural scripts of grief that shape how these women experience and express their losses remain largely invisible to institutional care.

In the context of displacement, ambiguous loss may also include non-human losses and identity-related losses connected to one’s homeland and aspects of the sense of identity and belonging to a place of origin [29]. From a psychosocial perspective, coping with this set of losses depends on the individual and contextual resources available. For many, the migratory process becomes a profoundly stressful experience, whose emotional, physical, and symbolic demands exceed their adaptive capacity [30].

Despite the importance of these losses as an inherent consequence of migration, research has paid scant attention to their symbolic and cultural dimensions, particularly in women from the Global South [31]. Migrant women are often approached from a needs-based or vulnerability framework, without fully exploring the meaning-making processes through which they interpret their losses and reconstruct their identities.

Understanding how migrants perceive and process these losses requires integrating a culturally informed perspective. Culture plays a crucial role in how individuals perceive and manage health and illness [32]. Arthur Kleinman [33] emphasises the importance of explanatory models, that is, personal and culturally shaped interpretations about the cause, course, and treatment of illness. He also highlights the importance of cultural and personal narratives in understanding human suffering and illness experience.

Beyond individual and cultural factors, it is essential to consider social determinants that structure migrants’ living conditions. The World Health Organization [34] underscores that access to education, housing, employment, and healthcare services is fundamental for health equity. In Spain, bureaucratic obstacles, legal precarity, and the lack of culturally sensitive care further aggravate symbolic losses and hinder adaptive coping [35]. In this regard, Madeleine Leininger’s Transcultural Care Theory [36] advocates for healthcare professionals to recognise and respect cultural differences, promoting care tailored to the values and beliefs of each patient.

From this conceptual framework, the objective of this study was to explore the vital losses experienced by migrant women based on their narratives and experiences, identifying key dimensions of these losses and the coping strategies emerging in relation to their health and well-being.

## 2. Materials and Methods

### 2.1. Design

This study employed a qualitative methodology, utilizing an interpretative phenomenological analysis (IPA) approach, as outlined by Smith et al. [28]. This approach allows for an in-depth exploration of participants’ subjective experiences, focusing on the interpretation of the meanings they attribute to their lived experiences. The choice of IPA was driven by the need to understand individual perspectives within their contextual and experiential framework, enabling a rich understanding of the phenomena studied through detailed and reflective analysis of participants’ narratives.

This methodological approach does not require a large number of questions but rather encourages an open, flexible, and in-depth exploration of participants’ narratives. Therefore, we started with the following questions: Can you tell me what this migration process has been like for you? What significant losses have you experienced since leaving your country? What did it mean to you, and how do you feel about it now? The interviewer adapted her approach according to each participant’s responses, using strategies such as silence, reformulation, or exploratory prompts (e.g., “Can you tell me more about that?”). These questions were used flexibly, depending on the pace of the conversation and the emerging content, thus fostering the co-construction of narrative and meaning, as proposed by the hermeneutic logic of IPA.

### 2.2. Participants and Sampling

A purposive sampling strategy was employed. Inclusion criteria required participants to be adult women, applicants for international protection, originally from Latin America, and residing in the southwest of Spain. Participants were contacted through a non-governmental organization working with this population. The sampling was designed to explore diverse yet comparable narratives among Latin American women seeking asylum, considering variables such as country of origin, age, and family status. Saturation was considered to be reached when no new experiential themes or interpretative nuances emerged that were relevant to the research objectives. This decision was grounded in an interpretative logic aligned with IPA, where saturation is not understood in terms of thematic repetition but rather as the point at which the analysis achieves a coherent integration of meaning while preserving individual nuances. As Elliott, Fischer, and Rennie [37] suggest, qualitative research may be considered sufficiently complete when it presents a dense, coherent, and persuasive narrative that honours the complexity of the phenomenon under study.

A total of 17 key informants participated. Seven semi-structured interviews were conducted to capture individual subjective experiences. Subsequently, two focus groups were held with five participants each to explore shared and social dynamics influencing meaning-making in the migratory context. Although IPA is traditionally idiographic, recent developments support the integration of focus groups in studies where meaning-making is embedded in relational and cultural contexts. As different studies suggest [38,39,40,41], group settings can facilitate the co-construction of meaning when participants share common cultural scripts, as in the case of migrant women navigating collective loss. In our study, the use of focus groups aimed to capture these shared frameworks while maintaining attention to individual voices through careful moderation, reflective questioning, and individual-level coding within group discourse.

In order to preserve the idiographic depth inherent to IPA, the analysis considered individual voices within group interactions, avoiding simplification into homogeneous discourses and allowing the capture of each participant’s particularities.

To address potential performative responses within group dynamics, the interviewer actively encouraged divergent views, clarified that there were no “correct” answers, and followed up on minority or contrasting perspectives. The analysis preserved individual contributions by assigning codes per speaker and comparing group interactions to individual narratives. This approach sought to ensure authenticity and reduce the influence of group norms on personal expression.

An informed consent letter was read aloud, and a copy was provided to all those who agreed to participate. It included detailed information about the study, its purpose, the potential benefits and risks, that the interviews would be recorded, how the research team would handle the participants’ data, and the voluntary nature of participation, without negative consequences for those who declined. None of the invited participants refused or withdrew from the study.

### 2.3. Data Collection

Data collection took place between November 2024 and February 2025, with fieldwork conducted in a private room at the University of Huelva to ensure confidentiality. All interviews were audio-recorded with prior informed consent, ensuring minimal loss of non-verbal cues critical for analysis. Data collection was carried out by two female researchers in collaboration with an external intercultural mediator. Interviews lasted approximately 40 min, and focus groups around 60 min, all audio-recorded. Additionally, the interviewer took field notes to complement the recordings. Both interviews and focus groups followed an interview guide developed by the research team after a thorough review of the existing literature.

Throughout the data collection process, participants’ emotional well-being was prioritized. All women were informed beforehand that they could pause the interview at any time, choose not to answer specific questions, or withdraw from the study without providing justification or suffering any consequences. Interviews were conducted in the presence of an intercultural mediator, who provided linguistic, emotional, and cultural support. The mediator played a key role in facilitating communication and ensuring a culturally sensitive atmosphere, particularly when participants used emotionally charged or culturally specific expressions. While not involved in analysis, the mediator contributed to the co-construction of meaning in the interview setting. Reflexive practice was central to working with the intercultural mediator. For example, the research team noted that certain metaphors used by participants were difficult to interpret literally. In response, the team engaged in debriefing sessions with the mediator to explore these expressions’ cultural meanings and adjusted the interview prompts accordingly. These reflexive discussions also guided decisions about when to pause interviews for emotional support or how to navigate differences in communication styles. Thus, the mediator’s role extended beyond translation, shaping data collection in ways that enhanced cultural sensitivity and ethical engagement.

Additionally, intercultural mediators not affiliated with the research team were consulted to ensure both literal and cultural translation of the content.

In cases where signs of emotional distress were detected, participants were offered breaks, empathetic listening, and the option to terminate the interview. At the end of each interview, participants were given information about available health and social services in the area. None of the participants reported significant distress during or after the interviews; on the contrary, several expressed gratitude for having been offered a space to share their experiences.

### 2.4. Data Analysis

Data analysis followed the stages described by Smith et al. [42] for IPA: (1) intensive reading of each transcript to become familiar with the individual narrative; (2) initial coding of meaningful segments expressing experiences of loss, pain, or reconstruction; (3) identification of emerging themes, articulating patterns of meaning within each case; (4) connection of themes across cases, developing shared themes and subthemes while preserving the uniqueness of each voice; and (5) construction of an interpretative narrative, integrating verbatim quotes with a comprehensive reading of the attributed meanings.

Due to the idiographic nature of IPA, each transcript was analysed in its entirety before proceeding to the next. IPA encourages participants to describe and reflect on their experiences—they are interpreting and making sense of what they have lived, while the researcher simultaneously engages in an interpretative process to understand what participants convey. This is known as double hermeneutics: the researcher accesses the participants’ experiences through their accounts but also interprets them through their own experiential lens [42,43].

Data analysis was conducted by two researchers trained in qualitative methods, who maintained continuous discussion to enhance the credibility of the findings. Reflexivity was practiced through analytic memos, team discussions, and journaling during analysis to remain aware of researchers’ assumptions, especially around cultural differences and gendered narratives of migration. Atlas.ti 23.0 qualitative data analysis software (CAQDAS) was used to support the analytical process.

### 2.5. Rigor

In addition to adhering to the Consolidated Criteria for Reporting Qualitative Research (COREQ) [44], the criteria for ensuring trustworthiness outlined by Lincoln and Guba [45] were applied.

Credibility was ensured by involving researchers directly in data collection and analysis. Methodological triangulation was conducted through the use of semi-structured interviews, field notes, and focus groups. Furthermore, verbatim quotes were used to ground interpretations in participants’ voices, and member checking was conducted with some participants to assess the fidelity of the interpretations.

Transferability was maintained by providing detailed descriptions of the study, including characteristics of the researchers, participants, settings, sampling strategies, data collection, and analytical procedures.

Dependability was promoted by offering a thorough description of the methodology used. Additionally, an external researcher reviewed the research protocol, focusing on methodological aspects and study design, and was also responsible for verifying the findings.

Finally, confirmability was ensured through triangulation techniques, fostering researcher reflexivity via reflective reports and detailed justification of the study, and by including validation from the participants.

The research team, composed of nursing professionals with intercultural and gender perspectives, critically reflected on their positionality throughout the entire process. Their clinical and community-based experience shaped a heightened sensitivity to the losses and suffering faced by migrant women, allowing them to engage in listening from a place of respect, empathy, and ethical commitment. To minimize interpretative bias rooted in autobiographical perspectives, spaces for self-reflection were promoted, facilitating a respectful and horizontal approach that recognizes participants as knowledge generators.

### 2.6. Ethical Considerations

All ethical principles established in the Declaration of Helsinki [46] were upheld. Participants were informed about the study’s objectives, the methodology employed, the voluntary nature of their participation, and their right to withdraw at any time without any consequences. Informed consent was obtained from all participants prior to both the interviews and focus groups. Confidentiality and anonymity were ensured in compliance with the Spanish Organic Law 3/2018 of December 5, on the Protection of Personal Data and Guarantee of Digital Rights. The study was approved by the TEDOC_MIGRA_2023 Ethics Committee.

## 3. Results

The study involved 17 migrant women from Latin America. Most were between 29 and 39 years old and were married or in a stable relationship. The majority were mothers and had a medium-to-high level of education. This sociodemographic profile suggests a group with considerable personal resources, whose migration trajectories nevertheless have been shaped by vulnerability stemming from irregular administrative status and social precarity. The sociodemographic characteristics of the participants are presented in Table 1.

The analysis of the participants’ experiences allowed the identification of two central themes that structure the narratives of the refugee women involved in this study: (1) the migratory experience as an experience of cumulative loss, and (2) reconstruction processes and significant gains. Through an interpretative phenomenological approach, subthemes emerged that reveal the profound subjective impact of forced migration and the strategies employed by the participants to cope with this process.

### 3.1. The Migratory Experience as an Experience of Cumulative Loss

This first theme reveals a complex and ongoing process of loss that refugee women experience from an existential perspective. Migration is configured as an accumulative grief process that encompasses identity, emotional, relational, material, and social dimensions. Participants described the migratory experience as a profound rupture with their previous vital references, generating a persistent feeling of uprootedness and emptiness.

#### 3.1.1. Fragmentation of the Self and Destabilization of Identity

Several participants described a significant disruption in their sense of identity following migration. This disruption was often linked to the loss of meaningful social roles—such as being a caregiver, professional, or community member—and the impossibility of sustaining a coherent life narrative. The migratory experience led to an erosion of the sense of self, lived as a fragmentation between who they were, who they are now, and who they will never be again. It is an intimate and often unspoken loss that extends beyond material deprivation, affecting participants’ sense of identity and self-perception.

*“I am not the same person since I left my country. My whole image changed. I lost the sense of happiness. I only show part of myself, something I don’t feel. I wake up every day with a fake smile because I have a daughter, a daughter who screams not to be here.”*

(RE1)

This testimony reveals one of the consequences of forced migration: a biographical discontinuity that interrupts the life narrative, creating a split between emotional and social identity. This can result in a profound existential crisis, a loss of purpose in the face of accumulated losses.

*“I lost the meaning of life. I’ve lived through so much grief… sometimes I wonder why and for what I’m still here.”*

(RE3)

#### 3.1.2. Breaking Ties and Emotional Uprooting

Emotional suffering was described by many participants as a persistent and silent state. Despite maintaining an appearance of strength, many women expressed feelings of sadness, anxiety, and emotional exhaustion. The constant sense of vulnerability, the struggle to sustain self-esteem, and the disconnect between what they show and what they truly feel internally reveal a profound emotional imbalance.

*“I get emotional because I see something I don’t like. Or because I can’t afford something. Or because I don’t feel valued.”*

(RE7)

The lack of recognition and appreciation becomes an open wound that deteriorates their self-perception and affects emotional regulation. It is also reflected, as in the following testimony, in a hypersensitive emotional body, saturated, constantly on alert, and vulnerable:

*“So anything—a shout, a word, a look that wasn’t even intentional—and then you feel it, boom! Chaos!”*

(RE5)

*“My mom doesn’t know I slept on the street. There are many things you don’t tell your family so they don’t worry, because everyone back home is anxious about what’s happening. I left the country alone. I’m alone.”*

(RE14)

All of this highlights an emotional self-isolation used to protect their loved ones, which further intensifies loneliness and pain.

#### 3.1.3. Structural Vulnerability and Loss of Autonomy

The migratory context was described as an environment of mistrust and constant exposure. Participants indicated that they had lost the ability to form emotional intimacy with others, as well as the basic safety necessary to establish protective bonds.

*“I think one of the things that gets hit the most is self-esteem. You lose a lot. Your value, your dignity, even the sense that you matter. You’re at the limit.”*

(RE9)

The feeling of being “at the limit” suggests an emotional overflow where self-esteem lacks external anchors or internal validation. The constant erosion of trust inhibits the rebuilding of secure attachments and social support networks crucial for promoting resilience and gratitude. The loss of self-esteem is thus not merely cognitive but an affectively charged state reflecting a crisis in self-worth and identity coherence.

*“Here, you can’t trust anyone. You have to watch your back all the time. Even when someone smiles, you wonder if it’s real. It’s like living with your guard up constantly, like a wall you can’t take down. Who’s really going to care about me?”*

(RE5)

This declaration highlights how the loss of trust operates not only as a cognitive barrier but as a profound social and emotional fracture that underpins feelings of isolation and emotional fragility. In this context, mistrust becomes both a symptom and a perpetuator of precarity, since it obstructs the formation of protective relationships and reinforces a sense of social exclusion. From a healthcare perspective, this pervasive mistrust and erosion of self-esteem directly shape how participants engage with health systems and professionals.

Participants’ reluctance to trust extends to healthcare providers, whom they may perceive as part of the same structural environment that has failed to protect them. The inability to establish safe, empathetic, and culturally attuned clinical relationships not only limits access to care but also deepens emotional withdrawal and a sense of invisibility within the system. For healthcare professionals, this dynamic challenges the creation of therapeutic alliances and may lead to dialectical conflicts, underreporting of symptoms, or non-adherence to treatment.

All this is further intensified by job instability and economic precarity, cross-cutting factors that shape the daily experiences of many migrant women. The inability to access formal employment, bureaucratic barriers to regularizing their status, and dependence on social aid were described as experiences that reinforce feelings of uselessness and psychological exhaustion.

*“We lose our professional field, which makes the drop in self-esteem even worse. I’m a nurse in my country. I have experience caring for older people, but here they only want live-in workers. They don’t offer part-time or full-time jobs. Since I don’t have papers, it’s very hard to find decent work.”*

(RE1)

*“I feel frustrated. We studied to help our country progress. To be doctors, lawyers… and to give that up and leave the country, knowing how hard it is to get our degrees recognized, is complicated.”*

(RE10)

Taken together, these narratives reveal that the migratory environment acts as a relentless context of threat and instability that severely restricts opportunities for emotional connection validation and the reconstruction of a positive self-concept.

#### 3.1.4. Symbolic Invisibility and Cultural Exclusion

One of the most profound losses experienced by migrant women is the forced separation from loved ones, especially parents, children, and siblings. The inability to be present during critical moments such as illness or death generates deep helplessness and pain. This absence of physical presence—and the impossibility of participating in culturally meaningful farewell rituals—results in a form of disenfranchised grief, unrecognized by the host society and thus intensified by isolation and silence.

*“Migration has taken so much from me. I didn’t see my siblings born, I couldn’t be at my grandmother’s funeral, I couldn’t study what I wanted…”*

(RE2)

These absences mark not only relational ruptures but also a loss of continuity and potential selves, undermining agency and self-worth. The mourning extends beyond the people lost to include the person they might have become. From a healthcare perspective, recognizing these cumulative experiences of loss allows for a more compassionate, person-centred approach that validates the elements of grief that are not always visible or culturally acknowledged.

*“When I came here, I was very close to my family. And that gets lost, it breaks. I dream a lot about my mom. I miss my mother so, so much, really, and my father.”*

(RE3)

Dreams emerge as symbolic spaces of unresolved grief, reflecting an internal mourning process in the absence of collective acknowledgement. In these intimate narratives, participants strive to maintain connection and identity in displacement.

*“I had to endure the death of my brother. That added to the pain I already had from losing my family, from being far from my home, my son, my parents. And on top of that, they called me, and I had to see my brother dead, murdered, from a distance.”*

(RE3)

Witnessing death remotely, through screens or calls, may introduce a form of moral injury, evoking guilt, shame, and emotional dislocation. The participant is confronted with a helpless proximity: emotionally close but physically excluded. The technological mediation of loss, devoid of communal rituals, fractures the moral and narrative frameworks that support mourning, complicating emotional processing and closure. The inability to act, to protect or to mourn in person, disrupts the narrative logic that normally accompanies death, hindering the process of healthy or non-pathological grieving.

Acknowledging how grief is experienced across borders and silences is key to opening up space for meaning-making and emotional accompaniment, beyond clinical symptoms, and offering culturally responsive support.

The spatial and emotional distance denotes a liminal state, emblematic of migrant precarity, where the participant is physically separated yet psychologically entangled with traumatic events. This is a profound challenge to closure and meaning-making, perpetuating emotional distress.

Across narratives, participants described this as a form of ‘invisible mourning,’ where their sorrow remained largely unacknowledged by their host society. However, the narratives also suggest emerging resilience, as participants navigate and reconstruct meaning within constrained sociocultural and migratory contexts. These efforts suggest that, even in contexts of structural exclusion, the search for coherence, belonging, and biographical repair persists.

#### 3.1.5. Displacement, Exclusion, and Loss of Belonging

One of the most deeply felt dimensions of loss described by participants is the erosion of social and symbolic structures that previously anchored their sense of belonging. In the host country, the disappearance of familiar neighbourhoods, routines, and relational fabrics is not only a practical disruption but a form of collective pain, a grief shared across bodies and borders.

*“But I also feel a collective pain, because many people left their homes, their surroundings, had to change territories. So I suffer for all those people, which intensifies my own pain. Destroyed environments, I have no home, and people… But when there’s war, people take what’s not theirs. ‘This is mine, now it’s mine because I say so.’ And that creates a feeling of anger and frustration. Because not only do I lose my personal space, but also my family, my culture, my society, etc.”*

(RE9)

The feeling of not having a place of their own is expressed both physically and emotionally. The forced nature of the departure implies a rupture in agency, purpose, and community engagement, resonating with narrative disruption and exile as a biographical fracture. Similarly, many participants described home not as a geographic location but as a relational and affective space—now lost. The absence of home is not merely material but also existential.

The participants expressed a form of transpersonal grief—an empathic identification with others who have suffered similar losses. The loss of “home” becomes a symbolic condensation of multiple absences: territory, culture, security, and community.

Moreover, this condition of uprootedness is compounded by experiences of xenophobia and social rejection in the host country, which actively deny the possibility of reconstructing belonging.

*“You also lose confidence in yourself. It’s lost because there is so much xenophobia in many countries around migration. And it’s hard, not just as an immigrant, to leave your country, your environment, your comfort, and go to another country. And they… how can I explain? They take it badly, like… ‘What are you doing here, go somewhere else.’”*

(RE12)

Xenophobic attitudes undermine not only the individual’s sense of safety but also their self-image, challenging their right to exist and belong. The internalization of rejection corrodes self-confidence, reinforcing a liminal state of “in-betweenness,” typical of migratory precarity.

### 3.2. Reconstruction Processes and Significant Gains

Despite the accumulation of losses, the second major theme reflects how refugee women activate internal and relational resources to rebuild their lives in the host country. This capacity for agency is expressed in three interrelated dimensions: practical and symbolic reconstruction of life, anchoring spiritual and emotional resources, and vital learning that emerges from suffering.

#### 3.2.1. Reconstruction and New Beginnings

Several participants shared experiences of personal “reinvention.” Rebuilding their lives in a new country involves redefining goals, assuming new roles, and creating new expectations. This reconstruction takes place within precarious conditions but is also accompanied by hope and openness to new possibilities.

*“One of the gains I may have had is getting to know myself better, identifying my needs and emotions.”*

(RE2)

This testimony illustrates how the awareness of one’s emotional landscape can become a first step toward healing. In some cases, this led to a redefinition of motherhood, womanhood, or professional identity, marking the beginning of a new narrative of dignity and autonomy, even in contexts of high vulnerability.

#### 3.2.2. Anchoring Spiritual and Emotional Resources

Faith and spirituality played a central role as a source of strength. Some women expressed that their connection with God or higher forces gave them meaning, hope, and energy to endure adversity. This spiritual dimension is intertwined with a daily attitude of struggle and emotional resistance.

*“But God makes us stronger, and if we are standing here right now, it is thanks to God’s mercy. Because otherwise, truthfully, some things would destroy us, bring us down. But we are daughters of God, and the Lord looks upon us with mercy.”*

(RE12)

Participants described this spiritual resilience as an active force that helped them resist despair, maintain hope, and find emotional refuge during moments of pain and loneliness. Spirituality was identified as a key resource for restoring self-worth and solidarity and for maintaining meaning amid loss and hardship.

However, participants recognized that structural barriers such as racism, legal irregularity, economic precarity, and limited access to healthcare constrained their opportunities for personal growth and tested the sustainability of their spiritual coping. These chronic stressors formed an adverse context in which resilience was expressed, highlighting the complex interplay between individual strength and systemic challenges.

#### 3.2.3. Search for Meaning and Personal Growth

Participants described the suffering associated with migration as an opportunity for inner growth and moral reflection. Many emphasized the need to “work on the inner self,” develop emotional autonomy, and establish personal boundaries as essential steps in their learning process.

*“One of the things I’ve gained is becoming a better person. When we come here and go to a government agency to ask for help, not everyone should be put in the same box as people who just like to ask for things. We need to be empathetic, because many times I don’t know what’s going on inside the other person, and a single word from me could even push them to suicide. We’ve seen that happen with two of our peers.”*

(RE15)

This testimony articulates a relational ethics of care, born from shared vulnerability. The ability to connect her own suffering with the suffering of others, reveals a heightened attunement to emotional fragility and the consequences of indifference.

*“Ever since I left my country, I’ve started to see things differently. I want to become a better person every day because I don’t know what might happen tomorrow. Just because we’re in a first-world country doesn’t mean we’re exempt from hardship, like what happened during the DANA in Valencia.”*

(RE15)

Migration here acts as a rupture that enables new modes of perception and connection. The testimony suggests that moral learning can emerge from adversity—but not without cost. These insights challenge simplified narratives of resilience, instead pointing to a complex moral labour of meaning-making, grounded in social injustice and emotional exposure.

*“You learn to be humble. Whatever you do, if you do it with love—and that’s what it’s about—bringing that love to people. Sometimes people look at it with suspicion, like you’re doing it for some hidden reason, but really, it’s just the pure intention of giving love to those who are willing to receive it. And even if the job seems very humble, there’s a lot of love being given.”*

(RE4)

This second narrative reinforces the idea of emotional agency—the ability to act with compassion even in undervalued or precarious social positions. Together, these accounts reveal that the search for meaning is not an individualistic journey but a deeply social and ethical process. At the same time, this calls for structural awareness, ensuring that systems of care do not rely on personal strength alone but actively support the conditions that allow such growth to occur with dignity, safety, and recognition.

## 4. Discussion

The findings of this study highlight the critical role of personal narratives as both therapeutic tools and forms of resistance for migrant women in vulnerable situations. Sharing their stories enables these women to reconstruct their identities and regain agency; as one participant stated, “*Sharing my story helped me feel seen and regain control over my life*” (RE4), consistent with Eastmond’s findings [47]. Through narration, women actively transform their subjectivity, giving voice to experiences often silenced or marginalized.

Migration entails multiple, interconnected losses—including identity, relational ties, material resources, and symbolic meanings—that profoundly affect mental health and emotional well-being. Participants described feelings of invisibility, worthlessness, and loneliness. As one shared, “*I felt invisible, like I had lost myself*” (RE8), echoing Bhugra and Becker’s [32] assertion that forced migration disrupts the symbolic structures underpinning continuity and belonging. These cumulative losses, exacerbated by legal insecurity, poverty, and discrimination, contribute to persistent psychological distress [48,49,50].

Mental health approaches must therefore incorporate compassion-based, culturally sensitive interventions that address structural barriers such as language, bureaucracy, and racism, which impede access to care [51,52]. A social identity perspective [53] elucidates how migration ruptures connections to family, community, and culture, destabilizing both individual and collective identities. This identity disruption aligns with Rosenberg’s self-esteem theory [54], as loss of professional roles and caregiving capacities undermines personal worth and social function [55].

Emotionally, participants experienced “migratory grief” [56], characterized by sadness, guilt, anxiety, and uprootedness, often silenced due to fear or shame. As several participants noted, “*here you can’t trust anyone*” (RE5). Attachment theory [57] further explains how separation from significant others weakens emotional security and trust, while the erosion of social networks increases isolation and distrust, even within migrant communities [58].

Economic and occupational losses also emerged as major vulnerabilities, with participants facing professional devaluation and precarious employment; this was particularly pronounced among refugee women confronting intersecting disadvantages of gender, ethnicity, religion, and legal status [59,60,61,62,63,64]. Despite these challenges, many women demonstrate resilience as a dynamic, contextually embedded process supported by social, cultural, and spiritual resources [65,66]. This resilience manifests through mutual support, spiritual practices, the reframing of migratory experiences, and an orientation towards gratitude and growth, reflecting post-traumatic growth theories [67]. One participant encapsulated this when she stated, “*A loss carries a gain. I have learned to become a better person*” (RE10).

Culture plays a pivotal role in shaping coping mechanisms, with religious and spiritual frameworks providing meaning and communal belonging [68,69]. Such culturally situated strategies have important clinical implications. Health professionals must adopt culturally sensitive and compassion-based approaches that recognize the emotional and symbolic dimensions of migratory grief [70]. This means creating safe, non-judgmental spaces where women feel heard in their experiences of loss and spiritual struggle. Narrative-based interventions—such as guided storytelling or reflective dialogue—can help process invisible grief and rebuild identity. Incorporating respectful spiritual inquiry into care and ensuring intercultural training for professionals are key to promoting trust, empathy, and culturally attuned support.

This study also invites a critical reflection on how vulnerability is conceptualized: it is not an inherent trait but a relational condition shaped by power asymmetries, institutional neglect, and social exclusion, which render some forms of suffering visible and others invisible [71,72]. Intersectional frameworks are essential to understanding gender-specific refugee traumas—such as gender-based violence and reproductive autonomy loss—which are often overlooked in clinical settings [73].

From a capabilities approach, migratory grief reflects not only psychological suffering but structural injustice, encompassing the erosion of fundamental freedoms such as choice, social participation, and recognition [74]. Understanding this complexity is vital for developing interventions that transcend symptom management and promote social repair, justice, and epistemic inclusion. Recognizing migrant women as active agents whose life stories and coping strategies embody resistance and hope is central to this endeavour.

## 5. Conclusions

This research offers a comprehensive perspective that weaves together the psychological, social, and cultural dimensions of migratory grief in women who have been forced to leave their countries of origin, emphasizing their situation of vulnerability. The findings reveal that the multiple losses associated with migration—identity, emotional, symbolic, and material—as well as the inherent suffering may lead some participants to develop coping mechanisms such as presence, gratitude, and spirituality. However, these should not be interpreted as inherently positive outcomes but rather as adaptive responses shaped by the absence of structural support and the need to navigate adverse conditions. These tools enable women to reframe their migratory experience and rebuild their identity within contexts marked by precariousness, invisibility, and exclusion.

The results highlight the urgent need to design health and social interventions that go beyond addressing material needs to also include emotional support, the recognition of the spiritual dimension of suffering, the promotion of community-based support networks, and the practice of compassion. Understanding and valuing these forms of resistance and re-signification can help guide practices toward more humanizing, collective, and empowering models of care.

In contexts of high social vulnerability, fostering spaces where the value of storytelling, community bonds, and cultural strengths are recognized may prove more transformative than approaches focused solely on clinical or individual dimensions. Listening to these voices and translating their experiences into concrete actions not only humanizes care but also redefines the ethical horizon of caregiving in increasingly diverse and complex societies.

### 5.1. Researcher Reflexivity

Given the sensitive nature of the topic and the participants’ vulnerable social situation, the research team engaged in a continuous process of reflexivity throughout the study. The team was composed of two Spanish nurses/researchers and one Brazilian researcher, all with prior experience in working with migrant populations and addressing issues related to health inequity, social exclusion, and intercultural care. This background facilitated empathetic listening, trust building, and cultural sensitivity during the research process.

At the same time, we acknowledge that our professional roles, academic training, and citizenship status—particularly as European and South American researchers conducting interviews with asylum-seeking women in vulnerable circumstances—may have influenced how questions were framed, how narratives were constructed, and how meanings were interpreted. For example, during the analysis of interviews, we modified the initial guide to include questions emerging from emotionally charged narratives, seeking to explore in greater depth topics initially not considered, such as spirituality and identity loss. Our own cultural perspectives and social positioning were critically examined throughout the study.

To mitigate potential biases and power imbalances, an intercultural mediator participated actively not only in data collection, contributing to the creation of a safer and more equitable interview space, but also in the analysis phase. Their cultural knowledge helped interpret specific terms and emotional expressions that might otherwise have been misunderstood or overlooked, enriching the understanding of participants’ narratives and ensuring a more contextualized reading of their experiences.

Additionally, field notes and reflective journaling were used to explore our positionality, emotional responses, and the assumptions we may have brought into the research. This reflexive stance aimed to foster ongoing critical awareness of the ethical and relational complexities inherent in cross-cultural qualitative inquiry.

### 5.2. Strengths and Limitations

A key strength of this study is its use of Interpretative Phenomenological Analysis (IPA), which enabled an in-depth understanding of how women seeking international protection make sense of their experiences of migratory grief. This approach facilitated the exploration of complex emotional, cultural, and existential dimensions, often overlooked in quantitative or purely clinical models. Giving voice to women who inhabit particularly precarious legal and social conditions contributes to filling a significant gap in the literature and foregrounds the lived realities of forced migration from an emic, meaning-centred perspective. The study also highlights the active role of these women in constructing resilience and meaning through collective practices, care, and spirituality, offering valuable insights for culturally competent and trauma-informed care.

However, some limitations must be acknowledged. Although the sample is small, this aligns with the characteristics of Interpretative Phenomenological Analysis (IPA), which prioritizes depth and richness of analysis over statistical generalization. This methodology allows for a detailed exploration of the participants’ subjective experiences and the meanings they attribute to them, which is particularly relevant in contexts of high vulnerability.

Nevertheless, the transferability of findings may be constrained by the specific sociopolitical context in which the study took place, as well as by linguistic, religious, and cultural factors that shape migratory experiences and expressions of grief. Furthermore, although reflexive strategies such as journaling and field notes, alongside researcher triangulation, were employed to strengthen the credibility of the analysis, the results are inevitably shaped by the researchers’ interpretations. This interpretation was later re-evaluated in team discussions, where cultural context and power dynamics were reconsidered, leading to a more nuanced understanding. Similarly, after early interviews, the research team revised the interview guide to better reflect participants’ non-linear migration trajectories and to avoid imposing Western assumptions about temporality and grief processing.

The intercultural mediator played a critical role beyond linguistic translation during interviews. She was consulted during the analysis phase to clarify the meaning of culturally embedded expressions, emotional gestures, and pauses. For instance, when several participants referred to “sadness” in describing their daily state, the mediator helped contextualize this term as a socially accepted way of expressing deeper emotional suffering, including fear and hopelessness. Her input directly influenced the interpretation of key themes and contributed to a culturally sensitive analysis. Future research could benefit from incorporating more culturally and linguistically diverse profiles, as well as longitudinal approaches to explore how the meanings of migratory grief evolve over time.

Additionally, participatory or co-produced research designs could empower communities and enhance the relevance and impact of findings. Integrating these approaches would support greater community ownership and contribute to policy translation, ultimately fostering more effective and just interventions.

## Figures and Tables

**Table 1:** Socio-demographic characteristics of participants (N = 17).

Pseudonym	Age	Country of Origin	Marital Status	Children	Educational Level ^1^
RE1	38	Venezuela	Divorced	No	Higher
RE2	19	Colombia	Single	No	Medium
RE3	37	Perú	Married	Yes	Basic
RE4	40	Nicaragua	Divorced	Yes	Higher
RE5	22	Colombia	Single	No	Higher
RE6	33	Cuba	married	Yes	Basic
RE7	36	Venezuela	Married	Yes	Basic
RE8	47	Perú	Married	Yes	Medium
RE9	28	Nicaragua	Married	No	Medium
RE10	44	Venezuela	Divorced	Yes	Higher
RE11	31	Venezuela	Married	No	Basic
RE12	24	Colombia	Single	No	medium
RE13	33	Perú	Single	No	Higher
RE14	42	Cuba	Married	Yes	Basic
RE15	45	Nicaragua	Married	Yes	Higher
RE16	39	Colombia	Divorced	Yes	Medium
RE17	53	Venezuela	Married	yes	medium

^1^ Educational levels were categorized as Basic (primary or lower secondary education), Medium (upper secondary or post-secondary non-tertiary), and Higher (tertiary education).

## Data Availability

Data supporting this study are available from the corresponding authors upon reasonable request.
